# Strategic collaboration councils in the mental health services: what are they working with?

**DOI:** 10.5334/ijic.838

**Published:** 2013-03-08

**Authors:** Andreas Liljegren

**Affiliations:** The Vårdal Institute, Department of Social Work, University of Gothenburg, Sweden

**Keywords:** strategic collaboration, mental health, social work, observation

## Abstract

**Introduction:**

In recent years collaboration has become an important part of the delivery of welfare services. One response to these collaborative efforts has been the introduction of strategic collaboration between different welfare agencies. Strategic collaboration is arguably the most open-ended form of service integration, as both purpose and membership are open to negotiation. This article will examine the work in strategic collaboration councils in the mental health services.

**Method:**

The study is based on observations in eight strategic collaboration councils in Sweden. The councils were observed over 12 months, and every meeting that was held during that time was observed and tape-recorded.

**Results:**

Four basic activities were identified: the exchange of information, the identification of problems, organizing events and activities, and organizing the councils. Even though these activities were identified, the main focus was to exchange information. The councils’ work also varied in terms of how they make decisions and agreements, and whether their focus is more on internal or external issues.

**Conclusion:**

From the identified activities, the councils can be classified into four ideal types: the information council, the problem-identification council, the decision-making council, and the self-organizing council.

## Introduction

The coordination of different mental health and social services has long been in the spotlight in the Swedish context for several reasons. The general deinstitutionalization of Swedish mental health care has meant that some mental health patients have gone from having one mental health care provider to receiving help from several organizations. As attempts are made to integrate these patients into society it becomes more important for the human service organizations to coordinate mental health services. Initiatives to (re)habilitate psychiatric patients outside of mental health institutions have drawn media attention to mental health care; so, too, have several violent crimes perpetrated by psychiatric patients, the prime example being the murder of Swedish Minister of Foreign Affairs Anna Lind in 2003. The subsequent government-initiated evaluation of the mental health care system concluded that more collaboration was needed [1]. The Swedish government invested €70 million in one of Sweden’s largest socio-political initiatives to promote integration of mental health services [2]. One expression of these ambitions comes in the form of managerial mental health councils. Even though the state has encouraged and demanded increased integration between the agencies concerned with mental health, no designated tasks have been assigned to these councils. Instead, it is left up to the councils to decide on who should be offered membership and what they should work with. It is not compulsory to have these kinds of institutions, and in some areas there are no councils of this kind.

Strategic networks represent a special way of organizing and governing the public welfare services. These kinds of networks have several traits that make them interesting to study. Networks are horizontally organized and stand beside the ordinary organizations. They are non-hierarchical, with decisions made by several actors of equal authority in what may be described as a pluricentric system, rather than having a single strong actor as the main decision-maker [3]. In more practical terms this means that the councils are very self-regulating and that the definition of their purpose, tasks, membership, and organizational structure are open to negotiation. Anyone can, for instance, decide that they do not want to participate; there are no formal sanctions that can force them into the network. In addition, the transparency of the networks is often limited. All of these conditions for organizing the councils make the question of what the councils do a very open one. For this reason it is also interesting to examine the interactions that occur in the councils. To answer the questions of what councils do, observations have been conducted in eight strategic mental health collaboration councils in three regions in Sweden.

Swedish mental health services offer highly specialized and professionalized services divided between authorities at the national, regional, and local levels [4] (Table 1). The organizations are managed at these levels, but are all represented at the local level. At the national level there are actual agencies that operate in Sweden. At the regional and local levels, various types of organizations operate.

Two groups of street-level workers have a special role in the coordination of services mentioned above: the personal ombudsmen and the case managers. They are not members of the councils but are still important in the provision of integrated care. The role of the personal ombudsman is to represent clients in relation to different agencies [5, 6], and the case manager has a coordinating function between the agencies.

The councils in the study are organized in two ways; they may be formally free councils or they may be included in a hierarchy of councils. Some are situated in small municipalities of about 10,000 citizens, and others are in cities with several hundred thousand citizens. The councils generally meet four times a year. The managers come from different levels in the organization, but some organizations are represented by someone from the operational level. In several councils there are also persons with some coordinating function either for the work of the council or for the work with mental health issues in the municipality. In some councils there are also representatives of local user organizations. In all, 57% of the council members are managers and the others belong to other groups. The dominant professional group representing the municipalities is composed of various kinds of trained social workers, and health care is dominated by medically trained staff, mainly nurses. Staff in the national agencies, such as the National Social Insurance Agency and the Swedish Public Employment Service, have more diverse professional backgrounds, most of them holding academic degrees in social science.

### Aim and research questions

On the one hand there is an increased trust in network governance but on the other hand it is possibly the most open ended form of attempt to integrate the services as both purpose and membership is opened to negotiation. The aim of the article is to describe and analyses how the members of the councils collaborate, and it examines the interactions occurring. How and to what extent do they integrate their services?

## Theory and methods

A very long-term process in the history of the division of labour is a move towards a higher degree of specialization [7]. One of several factors in this process has been the professionalization of different occupations [8–10]. It is well known that specialization demands higher degrees of (re)integration between the agents in a specific organizational setting, such as the provision of mental health services. While the organizations do have to operate as autonomous units in order to accomplish certain tasks, there are several significant advantages to their reaching out to other organizations, for example, organizations can cooperate to conserve resources and to negotiate roles, meanings, and identities [11]. Organizations and organizational units can be integrated both vertically and horizontally, and several forms of integration have been identified [12]. *Cooperation* involves high levels of both forms of integration. *Coordination* is primarily a vertical integration between units on different levels inside the organizational structure and is directed from higher levels towards lower levels. Another form of integration is *collaboration,* which is mainly a horizontal integration between units at the same level. Weaker forms of integration might be described as consultation [12]. Collaboration is the focus of the present study and “generally involves the exchange of resources or joint pursuit of mutual goals” [13 p. 7]. Inter-agency collaboration can take place on several levels in the organizations, from the street-level bureaucrats to the managerial level, which has a more strategic responsibility. Key agents in strategic collaboration are the managers who interpret and/or address the demands of a more integrated provision of services [14, 15].

Just as collaboration can take place at different levels in the organizations, ranging from the operational to the strategic levels, so can research. Research on collaboration at the operational level can focus on the interaction between agencies and the client/patient, or it can focus solely on interactions between the professionals [16–20]. A much less studied phenomenon is when the managers collaborate at the strategic level [21, 22]. Another observation that can be made is that there is a strong methodological focus on interviews and surveys. Some of these studies have focused on the effectiveness of collaboration and how to measure it. Nylén [4] concludes that the right combination of formalization and intensity has a high potential for increasing effectiveness. Trute et al. [23] conclude that more service coordination reduces the need for support over time. Ljung [24] concludes that local cooperation agreements are not positive for the integration of services. Åhgren et al. [25] constructed a model for evaluating collaboration that focuses on the service user and level of integration between the welfare services, and Smith and Mogro-Wilson [13] found that staff members are better predictors of collaboration than administrators. Others have focused their research on what enables and hinders collaboration. Enabling factors can include a functioning feedback system [15], joint coordinators [16], intentional and evolutionary processes within and between organizations [26], shared goals and a common vision of collaboration [27], and personal factors [18]. Despite what seems to be a general lack of observational studies of strategic collaboration, some studies have been done on child protection which have covered leadership issues and how strategic councils handle diversity [28, 29]. Some authors have also questioned how much is accomplished by such [30, 31]. (More is said about the lack of observational studies in the next section.)

The study was conducted in eight strategic collaboration councils in the northern, southern, and western regions of Sweden, and data were collected from December 2008 to September 2010. The selected councils had to meet four criteria. They had to:

- work with mental health issues
- be strategic; in other words, they had to be managerial councils
- be situated in one of our three selected regions
- be selected from a variety of municipalities, from small to big

The selected criteria do not make it possible to give the full picture of strategic collaboration councils in general. Instead, the goal is to give examples and show the complexities of the studied phenomena. The first two criteria had to do with the actual purpose of the research project, to examine the interaction occurring. The third and fourth criteria were introduced to get variation in the material. To capture any local variation among different councils, three regions were selected—the western (n:4), southern (n:2) and northern (n:2) regions of Sweden. In addition to capture variations due to the different sizes of cities, the councils were selected in three groups: those in small cities (<50,000 citizens, n:3), those in midsized cities (50,000–150,000, n:3), and those in large cities (>150,000, n:2). The councils were observed over 12 months, and every meeting that was held during that time was observed and tape-recorded. The observations were open-ended in order to “...grasp what the world looks like to the people...” of the councils [28 p. 218].

Each of the councils held between 3 and 6 meetings during the studied period; in all, 38 meetings were recorded. Most meetings lasted about 2 hours, but in some cases they took up to 6 hours, so the analysis is based on 82 hours of recordings. All recordings were transcribed and the data were analysed using the computer program Nvivo 9. Initially all data were read and the four researchers started to develop a thematic structure using the theoretical framework, focusing especially on the activities in relation to the perspective of integration but also in the light of the aim of the study, to describe and analyse how the councils collaborate. After the thematic structure was made, the material was divided into four parts and distributed among the researchers to do the actual coding. After another set of discussions on the coding the author reread all of the material in order to validate and deepen the analysis.

As noted above there is a strong tradition of studying collaboration through interviews and surveys. The advantage of these methods is that they are effective in highlighting how different actors *perceive* collaboration. However, how people with a vested interest perceive collaboration does not necessarily describe what *actually* happens. The connection between actors’ self-understanding and what they are really doing can be expected to be stronger when there are strong normative expectations, such as expectations of collaboration [32, 33]. As these organizations are expected to collaborate, it is also important for researchers to describe and analyse what *actually* happens in the course of strategic collaboration.

Finally, a note on citations. In order to preserve the confidentiality of the study participants, the councils have been coded from 1 to 8, but the citations have been connected to the position and the organization the member represents.

## Results

### What do they work with?

#### Information

An activity that is very common in the strategic collaboration councils is the exchange of information. Even though all councils spend some time doing this, there are significant differences in the amount of time spent on it. Information exchange is practically the only activity undertaken by some councils, whereas others have a more mixed set of working tasks. Even though information sharing is prioritized, as it is something that all councils do, it is often removed from the schedule if there is a shortage of time. The exchange of information can be conducted in at least two ways; it can be done systematically, with council members taking turns to describe what happens in their organizations, or it can be done in a more fluid discussion. Even though councils vary in the extent to which they focus on the exchange of information, the types of information they present are similar.

Studying what the councils work with reveals a *standard package* of shared information (i.e. information that is exchanged often). This includes (1) positions, (2) reorganizations, (3) projects, (4) collaboration, and (5) education, and what can be described as more *optional issues* such as (6) the state’s influence on the organizations within the psychiatric services, (7) case managers and personal ombudsmen, and some information related to (8) clients and people-processing procedures.

Information sharing about changes in different (1) *positions* in the organization is very common. A representative in one municipality, for example, states:

On the 16th of February I changed tasks and responsibilities and I’m the new director of the reorganized and centralized Department of Social Resources and I am responsible for the municipalities’ social welfare resources for mental health, excluding some formal decisions that still are up to the specific social welfare offices in the municipality. (Manager of the Community Services, Council 6).

(2) *Reorganizations* constitute another common topic (as noted above), either of member agencies or the surrounding structure of the whole municipality. The extent of the reorganizations can vary, but two topics occurred frequently. First, due to the general financial crises during 2009 many municipalities had to cut back their budgets and consequently they changed their structures or discontinued some programs. Second, the government made changes to primary health care, which meant that private initiatives were promoted and increased during 2008–2009. Primary health care systems were decentralized and the number of providers increased.

Information was also exchanged about (3)* projects* that organizations were running (or were about to start).

I think this might interest you. We have started two, three new housing projects. One is for persons with mental disabilities. (Manager of the Municipalities Social Psychiatry, Council 6).

After this statement the council member referred to a shelter for homeless people and a housing project for drug abusers. The user representative is quite active in this subject, reporting about a lot of activities from their organization.

(4) *Collaboration* is a common subject that often emerges when information about projects is presented, as some of the projects are the result of collaboration. In addition to these operational projects, information about other collaboration councils is also shared. At times this information is about other areas of collaboration such as work with young people, but most often it is about other councils that handle psychiatric issues. Some participants are also members of the other councils and often refer to the work of these other councils.

Another theme is about (5) *education and training*.

We initiated two new educational projects last year. One is to raise the general level of competence for staff who have no formal training and who work with mental health issues. (Manager of the Municipalities Social Psychiatry, Council 2)

Aspects of education covered included identification of potential sources of funding, actual applications for funding, applications that have been granted, and training that has been provided to staff groups, such as cognitive behavioural therapy, motivational interviewing, and case management.

The subjects identified above can all be seen as part of the standard package, but there are additional subjects that seem to be optional. *Issues initiated by the state,* such as clinical guidelines from the National Board of Health or other nationwide policies, affect the psychiatric field. The clinical guidelines include how to treat schizophrenia and drug abuse. We cannot identify whether the councils take any specific action on these guidelines, but they do talk about them. What happens is that someone who is more informed than others about the specific guidelines gives a general description of what they contain. The councils also discuss how they handle these guidelines in some of their own organizations. In some cases they also share information about some joint discussions that have taken place in other settings. One council was asked to evaluate what the national guidelines on drug abuse could mean for these organizations, but the council suggested that a special group be initiated to do this work.

Two professional groups are a repeated topic of discussion. A growing number of professionals in the Swedish psychiatric field are *case managers and personal ombudsmen*. Both groups work with some of the most difficult and challenging clients, often those presenting both a psychiatric diagnosis and drug abuse. Consequently, these groups have special insights into how collaboration works in practice. They also have a special role in identifying collaboration problems between the organizations. Some of the councils have a formal connection to the case managers and/or personal ombudsmen, offering them some form of guidance. However, despite these formal ties, operational decisions about their work are made elsewhere.

Some of council members occasionally talk about *clients and operative people-processing procedures*.

The increase in clients applying for social welfare allowance is tough. It is increasing. But when it comes to children and youth in day and night care, the figures are decreasing. (Manager of a Social Welfare Office, Council 6)

In early stages of the collaboration process, for example when new members are introduced, this subject is covered more thoroughly. However, later, when the members know each other, this topic comes up less frequently and tends to be discussed only when specific operative changes are made. Examples of these subjects could be procedures, rules, regulations, and the law. They also include growing and declining client groups, opening hours, and the length of time people have to wait to get help.

#### The identification of problems

In one segment of the meetings, members of the strategic collaboration councils identify and discuss different problems; the problems may be specific or they may relate to the psychiatric field more generally. In all of these examples, the discussions did not lead to any specific actions or interventions. The problems were aired, but the groups took no further action on them.

An important role in identifying problems is played by the personal ombudsmen. Together with the user representatives who participate in some of the councils, the ombudsmen bring concrete problems to the table. They have special insights into how the clients are treated as they both hear the stories from the clients and attend many of the meetings with the clients. The problems discussed by the personal ombudsmen and the user representatives are of a more practical nature, more closely related to the psychiatric patients, such as issues concerning how to be respectful towards the clients.

…there is a headline [in the report] about problems with how the clients are treated, where all personal ombudsmen highlight shortcomings in how the clients are treated. The professionals do not listen to the clients enough. (Manager of the Community Mental Health Care, Council 1)

There are several examples of these problems. The *professionals’ style of communication* (both verbal and written) is not adapted to many of the psychiatric patients, and time limitations generally do not allow for the professionals to ensure that the clients understand exactly what they are trying to communicate. This lack of proper communication creates frustration and conflict, according to some of the personal ombudsmen.

Another problem that is discussed is *dysfunctional collaboration.*

We’ve had a man whom we have helped a lot with treatment for his drug abuse, but when he came home again no one was willing to become his doctor so now he can’t continue with his medication. The treatment chain is clearly not working. (Manager of the Social Welfare Department, Council 4)

The identified problems are mainly located between members of the council and an organization that is *not* represented in the council.

A recurring problem that is discussed in most councils concerns a major change made by the state that involved changing the boundaries between the National Social Insurance Agency and the Swedish Public Employment Service, moving many clients from one to the other. The National Social Insurance Agency is also at the centre of another recurring inter-agency problem, which has to do with how physicians should write doctors’ certificates. On formal grounds the certificates have been dismissed. There are also other, more general, collaboration problems such as concerns that agencies are not informing collaborative partners about what happens with mutual clients.

Other problems are related to *specific client groups* that the organizations struggle with Young people in particular are highlighted as difficult; in addition to being young they also hurt themselves, use specific drugs, or passively stay at home (these are referred to as ‘home-sitters’). Another type of problem has to do with a *shortage of certain resources,* such as professionals trained in cognitive behavioural therapy. Besides persons trained in CBT, the council members need housing and jobs suitable for the clients. They also need staff physicians. As mentioned above, we cannot see that any action has been taken to address the identified problems.

#### Organizing events and activities

These strategic councils are not oriented towards making decisions and agreements. Among the things they do organize, there is a strong emphasis on education and professional training.

We have been asked to organize the Psychiatry Day, or rather Psychiatry Days, as we have decided on two days. We have nine persons in the project group; the dates are set for the 10th and 11th of October, and one of our ideas is to show a movie with a psychiatric theme and have a discussion group afterwards. (Manager of the Social Psychiatry, Council 2)

The most common form of education is directed towards the general public, and several councils have an event called a ‘Psychiatry Day’ (or week) with movies with a psychiatric theme, panel discussions, and thematic lectures with professional experts or persons with some special experience with the theme. Some councils do the planning themselves and make all the preparations concerning theme, localities, etc., but it is more common to delegate the practical planning to a special project group which reports back to the council. One person from the council usually chairs the project. In several councils this event is a recurring point of discussion. As Psychiatry Days are annual events, at any given time the councils are either planning for the next event or evaluating the last one. Besides public education events, some councils organize various joint events for professional staff. At times, councils higher up in the collaboration hierarchy put educational events together. Where this occurred in one instance, the higher council organized the event and left it up to the studied council to determine who could come.

Topics for this training can include neuropsychiatric disorders, young adults, suicide, and suicide prevention.

Some of the councils also have a formal supervisory function in relation to the case managers and/or personal ombudsmen.

Okay, let’s move on. I would like to tell you something about what has happened with the personal ombudsmen since we met last time. You were there too, Mike; the number of cases are increasing, aren’t they? (Manager of the Community mental health care, referring to a manager in the local Social Welfare Office, Council 1)

Even though some councils are formally superior to the case managers and personal ombudsmen, the councils take a passive role, and their work is reduced to sharing information. In this their role is much the same as that of councils that have no formal connection to the case managers and ombudsmen. However, there is one exception to this information orientation towards the case managers and personal ombudsmen. In one of the councils the personal ombudsmen produced their annual report criticizing the collaboration in the municipality. The report was published on the municipality’s home page before the council could read it. In this case the council took a very active part in the personal ombudsmen’s work, deciding that quarterly reports should be written to the councils, specifying any criticism they had towards the organizations participating in the council. In addition, a statement on the report was to be written commenting on what was seen as an unfairly critical report being published without the criticized organization having the opportunity to correct mistakes.

#### Self-organizing

An important part of what these councils do is organizing themselves.

Ok, should we have a meeting in August as well? No, I don’t think so. The week after? By that time we have met and planned for Psychiatry Day. (Manager of the Community mental health care, Council 1)

Among the issues that fall under this category is the matter of *when and where to meet*. A recurring and difficult question to answer is when to meet, as most members have tight schedules and it is necessary to decide on meeting dates long in advance to enable as many as possible to attend. Even so, some members do get excluded from council meetings. Deciding on who those persons should be seems to be a difficult task, which takes some time. On the more practical side there are also discussions on *who should take the roles of chair and secretary*.

I recall that in the beginning we talked about changing chair and secretary between the municipality and the psychiatry professionals. If you want some other agency in these posts we can talk about it. (Manager of the Community mental health care, Council 1)

Several of the councils have a pattern of changing the chair and secretary on a rolling two-year schedule between the municipality and the community mental health care agency. In addition to these practical issues there are three questions that occupy the councils; these concern first, *who the council should include as members*, second, *what the council should do,* and third, *how the collaboration council should be reorganized*.

About the membership, some council members would like to see the National Insurance Agency and the Swedish Public Employment Service join the councils, but only two councils have succeeded in this respect. Some councils have the desired members but find it difficult to get their members to attend the meetings. The primary health care and the in-patient psychiatry professionals in particular prioritize other things ahead of the collaboration councils. In two of the councils the user representative does not attend. In one of these councils, the absence of the user representative is considered to be a big problem, which is discussed thoroughly; in the other council, the representative’s absence does not appear to be a problem. The primary health care representation is discussed for another reason. The question about mandate is raised. As primary health care is decentralized it becomes unclear who the public primary health care member represents in the council.

The second question some of the councils discuss is the *purpose of the meetings,* and this issue is most often initiated by someone asking for clarification on the actual purpose.

- But what, what is the purpose of this group? What kind of group are we? We do different things. Are we some kind of project group or are we a collaboration group or what?
- You could say that we are a collaboration group for managers. You could say that. (Manager of the Social Welfare Department, Council 3).

In this case there are no attempts to (re)negotiate the purpose of the council. Such a negotiation did occur in another council where a member suggested that the council should do more than share information. This question of purpose is especially an issue in the councils that are integrated in a hierarchical collaboration structure. The purpose can be both general and specific; that is, there is the general purpose of the council, and the specific purpose of a particular event, such as a Psychiatry Day. These questions can come from the council and be directed towards the council higher in the hierarchy, or they can come from the ‘leader council’ directed towards the councils that we are studying.

In three of the councils there were also considerable discussions about the reorganization of the collaboration structure. In one case the council members strongly opposed the reorganization as they were very happy with the work of the council. Later the suggestion that they reorganize was retracted and the work continued as before. The other two councils did not oppose the changes, but it took some time to inform them about the new collaboration structure. The explicit purpose behind the reorganization of two of the councils was to reduce the number of collaboration councils in which the municipality met with the region. In one of our studied councils the general collaboration hierarchy was revised and the number of councils was reduced from 70 to 7 (of which one covers mental health issues).

So far it has been shown that the councils are oriented towards the exchange of information and not so much towards making decisions and agreements. How, then, can the work of these councils be understood? How and to what extent do they integrate their services? It has also been shown that the council has four main activities oriented primarily towards the exchange of information. From the councils’ four main activities four ideal types of councils might be constructed: *the information council*, *the problem-identification council, the decision-making council,* and *the self-organizing council*. As shown above, *the information council* is concerned with sharing information about what happens in the organizations, along with other information that may be relevant to the councils. Information is an essential part of any collaborative activity and these councils prioritize this aspect of the collaboration. Any adjustment between the organizations or within a particular organization is entirely up the member(s), to be made independently of the council. This can be seen as a low level of horizontal integration. How much vertical integration/coordination the activities of the information council might lead to is an open question. In practice, the information aspect seems to be something of a paradox. Information sharing is the highest priority, with some councils spending all of the time they have together doing this, and it is a significant part of the sessions in all councils. However, information sharing is the first thing removed from the schedule when time is limited and the only time a council tries to renegotiate the agenda of the council meetings is in order to do things other than exchange information.

*The problem-identification council* goes one step further in the integration process and identifies different problems even though it does not take any shared action to address the problems. It is up to the members to do that outside the council. Even though no shared action is taken on identified problems, it does not mean that the agencies cannot make changes outside the council. The identification might lead to collaboration, cooperation or coordination, but both of these first two types of councils have such a low level of integration that it should be seen as a form of consultation. As in the case of the information council, it is an open question as to how much vertical integration this might lead to.

The *decision-making council* does all of the tasks mentioned above, and it also takes shared action on both information and problems. For instance, it can organize applications, hold different events such as educational events for staff or the general public, and negotiate organizational boundaries. In the case of the decision-making council, depending on the kinds of activities it organizes, there are higher levels of both horizontal and vertical integration, especially if the activities have consequences for the jurisdictional boundaries of the organizations. If the earlier forms of integration can be described as consultation, the decision-making council is a form of collaboration.

The last ideal type of council would be the *self-organizing council,* which primarily focuses on the council trying to set itself up to coordinate its internal activities in accordance with its purpose, membership, and mandate. The orientation towards the internal issues of the council can be seen as a sign of the initial phase of the collaboration process and/or as a sign of a transformation of the aim and/or membership. If the council focuses on these issues for a longer period of time it seems to indicate a lack of collaboration abilities, but it seems important to focus on these issues to some extent. It can also be seen as a precollaboration or preconsultation phase. Even though some integration is accomplished, this kind of council should rather be evaluated for what comes out of this initial phase. The activities in the eight councils can be summarized in the following way (Table 2):

As can be expected, the actual councils are to some extent a mixture of types, even though two of the councils are almost exclusively oriented towards the exchange of information. In some cases there is no or very little activity in some types categories. Minimal activity in the self-organizing category indicates that the councils do the necessary preparations, such as deciding on time and place for the next meeting, but there are no discussions on the purpose and membership.

## Discussion and conclusions

**Figure 1.**
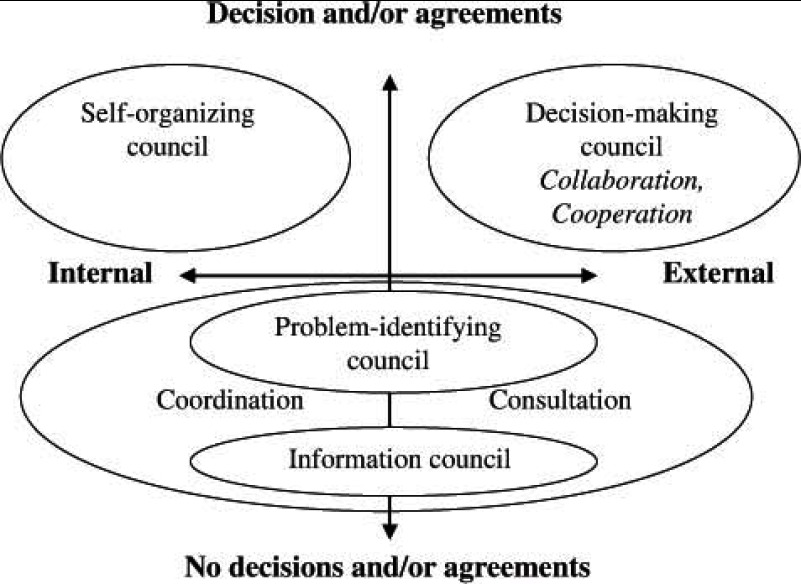
Ideal types of councils.

From a more abstract perspective, the activities of these councils can be described and analysed in relation to two dimensions: from no decisions to decisions, and from internal to external, creating a two-dimensional model (Figure 1).

In the lower part of the model no decisions or agreements are made, and this is where the information council is located. The information council can focus on internal issues, such as information from the participating organizations, and it can also look at more external issues, such as general guidelines or other issues related to the mental health area. Above the information council in the matrix is the problem-identifying council. This is closer to making actual decisions, but it still focuses on identifying problems, not actually solving them. These problems can also be internal or external. An example of an internal problem would be a lack of resources, whereas an external problem might stem from a non-participating organization, or from politics or policies. In the lower part of the model there are also lower degrees of integration and what can be described as two forms of consultation. The exchange of information and the identification of problems might also lead to the organizations coordinating their services internally.

In the upper part of the model are the councils that make decisions. On the upper left side is the self-organizing council. The concept of a council rests on some kinds of decisions being made about who should be a member, and for that reason the self-organizing council is located in the upper left side of the model, but the focus is on itself and its functions as a council. However, the decision-making council makes other decisions too, which can also focus on more internal or external issues. When it comes to the decision-making council it is also possible to describe it in terms of collaboration and/or cooperation. An internal issue might concern a shared educational event for staff, and an example of an external issue might concern a public education event. In this ideal typical construction of the councils it is argued that the information council is more oriented towards presenting information about internal issues, such as people-processing procedures. The decision-making council is more oriented towards sharing information about external issues, such as the socio-political context.

There seems to be no lack of coordination between the main actors, the municipality, and the regional mental health care service; rather, there are multiple forums for collaboration in the psychiatric field. This impression is supported by two observations: first, the frequency of referrals to other collaboration councils, and second, the fact that two out of eight councils were reorganized during the time we made the observations. In both of these cases the motive for the reorganization was to reduce the number of collaboration councils. Even though there seem to be plenty of opportunities for the main actors to meet, it is not certain how much they accomplish during the meetings. The lack of decisions and agreements may be seen as an indication that these councils do not accomplish very much, just as earlier research has shown [26, 27]. It might be tempting to conclude that the councils have the potential to make a significant contribution to mental health care, but the lack of actual decisions and agreements may be seen as a sign that the councils are not making such a contribution. This conclusion would be the one made in some of the earlier research on strategic collaboration [30, 31]. Even though it might be tempting to draw such a conclusion, it is important to remember that what goes on in the council can lead to members having contact with each other outside the council and taking action on matters discussed during the meetings. The value of these councils might not be found in the decisions at all; it is possible that their contribution lies elsewhere, such as in the opportunities they create for members to get to know other important actors, or the benefits may lie solely in the exchange of information.

In sum, the article makes at least four main conclusions/contributions. First, there are four types of activities in the councils: the exchange of information, the identification of problems, organizing events and activities, and self-organizing. Second, there are two organizing principles in relation to the councils: one is related to decision-making and the other is related to orientation towards internal or external affairs. Third, the councils observed in this study are more oriented towards information exchange, which might lead to questions about how much the councils accomplish—but the fourth conclusion is that this might be too hasty a conclusion.

A problem connected to participant observations is the effect that the researcher might have on what is observed. How the researchers’ participation may have affected the councils is difficult to say, but one of the chairs welcomed the researchers, remarking that “...it is a good way of getting everybody to turn up”. This can be interpreted as the researchers’ presence having an influence on the councils. A critical question might be asked in relation to the low level of managers in the councils (57%). Maybe the claim of being strategic reflects the ambitions of the councils more than it does the actual situation. The members might be inclined to exaggerate how strategic the councils are. At least two reflections can be made in regards to this. First, it is possible that the project has only partly succeeded in recruiting the type of council that the project meant to study. Second, the number of managers may reflect the actual situation for the council, in that it may be difficult to recruit members at the appropriate level.

What does this mean for the wider context of integration? The article highlights what strategic collaboration councils actually do and how that can be understood. The results highlight important normative questions on what strategic collaboration councils actually should do. Maybe it is too much to look for any easily identifiable benefits for the clients or any easily detectable forms of integration. Another question that might be asked is what recommendation might be made for the managers who are expected to be involved in strategic collaboration. The article offers a categorization of four types of councils and what they can work with. The recommendation would be to use the categorization to make the purpose and work of the council more explicit. The framework developed in this article could be a tool in that process. There could be at least two gains by this. First, for the internal work, to define what kind of council, with a shared aim, can give guidance in this work and avoid all the problems that are related to not sharing a common goal. Second, there seems to be an idea that the exchange of information or identifying problems is not worthwhile. To use the framework to specify and make the purpose explicit could also be a way to avoid the criticism that the council is not doing what it is supposed to.

## Reviewers

**Susanna Bihari Axelsson,** Associate Professor in Public Health, Aalborg University, Denmark.

**Bonnie R. Swaine**, PhD, School of Rehabilitation, Faculty of Medicine, Université de Montréal, 7077, Avenue du Parc, C.P. 6128 Succ. Centre-ville, Montréal, Québec H3C 3J7, Canada.

One anonymous reviewer.

## Figures and Tables

**Table 1.**
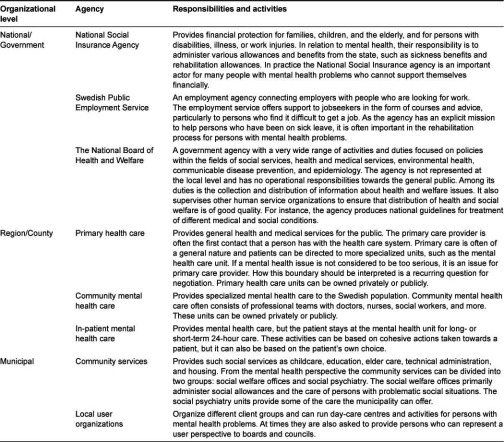
Organizational level, agent/agency, and responsibilities and activities

**Table 2.**
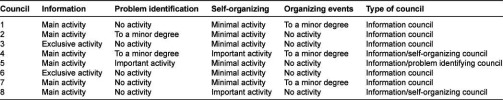
Summary of activities
